# COVID-19 Hospitalization in Hawaiʻi and Patterns of Insurance Coverage, Race and Ethnicity, and Vaccination

**DOI:** 10.1001/jamanetworkopen.2024.3696

**Published:** 2024-05-01

**Authors:** Brock M. Santi, Philip A. Verhoef

**Affiliations:** 1 Department of Medicine, John A. Burns School of Medicine, University of Hawaiʻi, Honolulu; 2 Hawaiʻi Permanente Medical Group, Honolulu

## Abstract

**Importance:**

The people of Hawaiʻi have both high rates of health insurance and high levels of racial and ethnic diversity, but the degree to which insurance status and race and ethnicity contribute to health outcomes in COVID-19 remains unknown.

**Objective:**

To evaluate the associations of insurance coverage, race and ethnicity (using disaggregated race and ethnicity data), and vaccination with outcomes for COVID-19 hospitalization.

**Design, Setting, and Participants:**

This retrospective cohort study included hospitalized patients at a tertiary care medical center between March 2020 and March 2022. All patients hospitalized for acute COVID-19, identified based on diagnosis code or positive results on polymerase chain reaction–based assay for SARS-CoV-2, were included in analysis. Data were analyzed from May 2022 to May 2023.

**Exposure:**

COVID-19 requiring hospitalization.

**Main Outcome and Measures:**

Electronic medical record data were collected for all patients. Associations among race and ethnicity, insurance coverage, receipt of at least 1 COVID-19 vaccine, intensive care unit (ICU) transfer, in-hospital mortality, and COVID-19 variant wave (pre-Delta vs Delta and Omicron) were assessed using adjusted multivariable logistic regression.

**Results:**

A total of 1176 patients (median [IQR] age of 58 [41-71] years; 630 [54%] male) were hospitalized with COVID-19, with a median (IQR) body mass index (BMI; calculated as weight in kilograms divided by height in meters squared) of 30 (25-36) and Sequential Organ Failure Assessment score of 1 (0-2). The sample included 16 American Indian or Alaska Native patients, 439 Asian (not otherwise specified) patients, 15 Black patients, 66 Chinese patients, 246 Filipino patients, 76 Hispanic patients, 107 Japanese patients, 10 Korean patients, 299 Native Hawaiian patients, 523 Pacific Islander (not otherwise specified) patients, 156 Samoan patients, 5 Vietnamese patients, and 311 White patients (patients were able to identify as >1 race or ethnicity). When adjusting for age, BMI, sex, medical comorbidities, and socioeconomic neighborhood status, there were no differences in either ICU transfer (eg, Medicare vs commercial insurance: odds ratio [OR], 0.84; 95% CI, 0.43-1.64) or in-hospital mortality (eg, Medicare vs commercial insurance: OR, 0.85; 95% CI, 0.36-2.03) as a function of insurance type. Disaggregation of race and ethnicity revealed that Filipino patients were more likely to die in the hospital (OR, 1.79; 95% CI, 1.04-3.03; *P* = .03). When considering variant waves, mortality among Filipino patients was highest during the pre-Delta time period (OR, 2.72; 95% CI, 1.02-7.14; *P* = .04), when mortality among Japanese patients was lowest (OR, 0.19; 95% CI, 0.03-0.78; *P* = .04); mortality among Native Hawaiian patients was lowest during the Delta and Omicron period (OR, 0.35; 95% CI, 0.13-0.79; *P* = .02). Patients with Medicare, compared with those with commercial insurance, were more likely to have received at least 1 COVID-19 vaccine (OR, 1.85; 95% CI, 1.07-3.21; *P* = .03), but all patients, regardless of insurance type, who received at least 1 COVID-19 vaccine had reduced ICU admission (OR, 0.40; 95% CI, 0.21-0.70; *P* = .002) and in-hospital mortality (OR, 0.42; 95% CI, 0.21-0.79; *P* = .01).

**Conclusions and Relevance:**

In this cohort study of hospitalized patients with COVID-19, those with government-funded insurance coverage (Medicare or Medicaid) had similar outcomes compared with patients with commercial insurance, regardless of race or ethnicity. Disaggregation of race and ethnicity analysis revealed substantial outcome disparities and suggests opportunities for further study of the drivers underlying such disparities. Additionally, these findings illustrate that vaccination remains a critical tool to protect patients from COVID-19 mortality.

## Key Points

**Question:**

Are insurance coverage, race and ethnicity, and vaccination associated with COVID-19 hospitalization outcomes in Hawaiʻi?

**Findings:**

This cohort study of 1176 patients hospitalized for COVID-19 found no association between type of insurance coverage (commercial, Medicare, Medicaid, uninsured) and hospitalization outcomes; however, significant disparities were observed among different races and ethnicities and at different time periods in the COVID-19 pandemic. Receipt of at least 1 COVID-19 vaccination was associated with significantly reduced risk of in-hospital death and intensive care unit transfer.

**Meaning:**

These findings suggest that efforts to expand insurance coverage and to understand the impacts of disease on disaggregated racial and ethnic populations should be important priorities, both in preparing for the next pandemic and for equitable distribution of health resources, such as vaccines.

## Teaser

This cohort study evaluates the associations of insurance coverage, race and ethnicity (using disaggregated race and ethnicity data), and vaccination with outcomes for COVID-19 hospitalization in Hawaiʻi.

## Introduction

While the COVID-19 pandemic has had a devastating impact on the US, Hawaiʻi had the lowest standardized death rates per capita in the country.^1^ Geography notwithstanding, understanding Hawaiʻi’s unique features may inform future pandemic preparedness and provide lessons for how other states can both improve their outcomes and target resources to individuals at the greatest risk of adverse outcomes.

According to US Census Bureau data, Hawaiʻi is the most racially and ethnically diverse state in the country, with 37.2% of the population identifying as Asian, 1.6% as Black, 10.8% as Native Hawaiian or Other Pacific Islander, 22.9% as White, and 25.3% as more than 1 race or ethnicity.^2^ Unfortunately, Asian, Native Hawaiian, and Pacific Islander populations are often considered as a single entity in the study of health outcomes, in spite of evidence that disease incidence may vary substantially among different racial and ethnic subgroups.^3,4,5^ However, while disaggregation of data can be challenging, several publications early in the COVID-19 pandemic suggested markedly disparate outcomes that merit further exploration.^4,6,7^

In addition, Hawaiʻi has the fourth lowest uninsurance rate in the country, largely due to legislation passed in 1974 mandating that employers provide health care to all employees working more than 20 hours per week.^8,9^ However, having insurance alone does not assure health, and there is a large body of literature documenting differences in outcomes for people with common diseases, such as cancer or sepsis, as a function of insurance coverage.^10^ For care of COVID-19 specifically, uninsured patients had significantly higher mortality and hospitalization rates, leading to calls for universal health care coverage as a component of pandemic preparedness.^11,12^

In this study, we evaluated hospitalizations for COVID-19 between March 2020 and March 2022 among patients admitted to a single institution, hypothesizing that the type of insurance (commercial, Medicare, Medicaid, or uninsured) would be differentially associated with outcomes. In secondary analyses, we used disaggregated self-identified race and ethnicity data to test the hypothesis that certain racial and ethnic subgroups may have fared worse when hospitalized with COVID-19, and we analyzed the potential association of COVID-19 immunization on outcomes.

## Methods

This cohort study was approved by the Kaiser Permanente Moanalua Medical Center institutional review board with a waiver of consent due to the use of deidentified electronic health record (EHR) data. This study is reported following the Strengthening the Reporting of Observational Studies in Epidemiology (STROBE) reporting guideline.

### Study Design

This hospital-based retrospective analysis used EHR data from the Kaiser Permanente Moanalua Medical Center in Honolulu, Hawaiʻi. All patients admitted between March 2020 and March 2022 with *International Statistical Classification of Diseases and Related Health Problems, Tenth Revision (ICD-10)* coding of U07.1 or positive results for SARS-CoV-2 in polymerase chain reaction testing were included in the study. Patients with multiple admissions were entered only once, with preference given to admissions directly related to acute SARS-CoV-2 infection rather than those associated with long-term sequelae or other primary diagnoses.

### Variables

Insurance status was classified into 4 groups. Commercial insurance includes both employer-sponsored private insurance or insurance purchased on exchanges and without any form of government-sponsored insurance. Medicare refers to insurance provided by the federal government for patients 65 years or older or with specific disabling conditions and includes any patient with either traditional Medicare A or B plans (which operate as fee-for-service insurance provided by the government) or Medicare Part C (also known as *Medicare Advantage*, in which the federal government pays private insurance companies a capitated rate to insure patients who qualify for Medicare). Medicaid refers to federally sponsored, state-administered health insurance for certain low-income patients, including children, pregnant women, and older adults, and includes any patient with exclusively Medicaid coverage (but not Medicare and Medicaid dual-eligible patients, who were counted as having Medicare). Patients with no insurance at the time of hospitalization were classified as uninsured. Of note, Medicare patients included patients with Kaiser Permanente Part C plans as well as Part C plans from other insurers. Similarly, Medicaid patients included those with Medicaid provided through the Kaiser Permanente MedQuest program as well as through other MedQuest insurers.

Comorbid illness conditions associated with an increased risk for severe COVID-19 according to the Centers for Disease Control and Prevention were identified based on *ICD-10* codes present in the EHR prior to hospitalization.^13^ To control for socioeconomic neighborhood conditions that may impact risk for COVID-19, we identified the proportion of households receiving public assistance and the proportion of households with below poverty level income within a patient’s census geocode using publicly available census data.^2^

Self-reported race and ethnicity are gathered at the time of enrollment for Kaiser Permanente insurance and at the time of presentation for clinical care among non–Kaiser Permanente members; these data were available in the EHR. Patients can self-identify from among the following American Indian or Alaska Native, Asian Indian or Pakistani, Black, Chamorran, Chinese, Fiji Islander, Filipino, Guamanian not otherwise specified (NOS), Hmong, Japanese: Kampuchean, Korean, Laotian, Melanesian NOS, Micronesian NOS, Native Hawaiian, New Guinean, Asian NOS, Pacific Islander NOS, Polynesian NOS, Samoan, Tahitian, Thai, Tongan, unknown, Vietnamese, or White. In addition, patients can self-identify as Hispanic or non-Hispanic ethnicity. Patients are also able to self-identify as multiple different races or ethnicities; for this study, all analyses were conducted using these self-identified categories, without post hoc grouping by the investigators.

COVID-19 wave data (pre-Delta, Delta + Omicron) reflect the period during which each respective SARS-COV2 variant was thought to be predominantly responsible for infections. Pre-Delta was defined as any hospitalization for COVID-19 before June 10, 2021. The Delta variant was first identified in Hawaiʻi at this time and resulted in a significant wave of infections starting in early July 2021, while the Omicron wave was responsible for hospitalizations from December 1, 2021, to the study’s conclusion in March 2022.

Vaccine status was classified as a binary variable based on available EHR data and included vaccines given within the Kaiser Permanente system and those received outside of the Kaiser Permanente system which patients reported to their health care practitioner (which were subsequently entered in to the EHR). A patient who received 1 or more vaccines of any manufacturer was categorized for these analyses as vaccinated. Analysis of vaccination status was limited to after June 10, 2021, when more than 65% of Hawaiʻi residents had received at least 1 COVID-19 vaccine (which the WHO had previously estimated may be adequate to achieve herd immunity), coinciding with the beginning of the Delta and Omicron waves.^14^

Body mass index (BMI; calculated as weight in kilograms divided by height in meters squared) and Sequential Organ Failure Assessment (SOFA) score were defined as a single value based on first available data after admission. Subsequent SOFA scores and BMI during the hospitalization were not included. Eight patients did not have BMI data available in the electronic record; these missing data were imputed based on the median BMI for that insurance category.

### Outcomes

The outcome intensive care unit (ICU) transfer included only patients treated by specialists in critical care within the ICU; patients who were transferred to a bedspace within the ICU (but did not require ICU-level of care as indicated by nursing ratio and intensivist involvement) were not counted for this outcome. Mortality was defined as a patient with discharge code 12020, indicating death during an admission; this was independently confirmed through manual EHR review. Patient death occurring in postacute care facilities or in the outpatient setting (after discharge) were excluded. SOFA score was used as both a variable of interest and an outcome measure.

### Statistical Analysis

For all analyses, statistical significance was established for 2-sided *P* < .05. For continuous dependent variables, analysis of variance was used to determine whether there were statistically significant differences among groups, while for categorical variables, χ^2^ analysis was used. Multivariate logistic and linear regression were used to analyze risk of hospital mortality and transfer to ICU. Given the well-documented associations between COVID-19 outcomes and age, BMI, or sex, all regressions were adjusted for these variables. In addition, regressions were adjusted for comorbidities and for the proportion of households within a patient’s census geocode who were receiving public assistance or below the federal poverty limit. Linear regression was used for all regressions with a continuous outcome measure (eg, SOFA score). We used logistic regression when both input variables and outcome measures were categorical (eg, vaccine status and hospital death). All regression analyses, including vaccine status, were conducted using a subset of the data that excluded patients admitted in the pre-Delta period, since most of these patients had not yet received vaccines according to available Hawaiʻi public health data. Because the self-identified race and ethnicity survey allowed for multiple answers, all analyses including the self-identified race and ethnicity variable were computed as univariate regressions to reduce multicollinearity. Regressions using insurance status were multivariate, as each patient received a single insurance classification. Statistical analysis was performed using R software version 4.2.2 and R Studio software version 2022.07.2 (R Project for Statistical Computing). Data were analyzed from May 2022 to May 2023.

## Results

Between March 2020 and March 2022, 1176 patients (median [IQR] age, 58 [41-71] years; 630 [54%] male) were hospitalized at Kaiser Foundation Hospital (Honolulu, Hawaiʻi) with COVID-19, with a median (IQR) SOFA score of 1 (0-2) and BMI of 30 (25-36) (Table 1). The sample included 16 American Indian or Alaska Native patients, 439 Asian NOS, 15 Black patients, 66 Chinese patients, 246 Filipino patients, 76 Hispanic patients, 107 Japanese patients, 10 Korean patients, 299 Native Hawaiian patients, 523 Pacific Islander NOS patients, 156 Samoan patients, 5 Vietnamese patients, and 311 White patients; races reported by fewer than 5 patients were not included. A total of 1165 patients (99%) reported at least 1 race, while 1043 patients (89%) reported 2 or more races. The plurality of patients in this analysis were commercially insured (Table 1). We observed associations between insurance status and sex, age, BMI, admission SOFA score, select races and ethnicities (Black, Chinese, Japanese, Korean, Pacific Islander NOS, Samoan), vaccination status, and in-hospital mortality (Table 1). The eTable in Supplement 1 shows the percentage of patients with comorbid conditions known to be associated with increased severity of COVID-19 as a function of insurance status. Medicare patients presented with higher SOFA scores and vaccination rates, as well as higher rates of multiple comorbidities and in-hospital mortality (Table 1). Patients who identified as Chinese, Japanese, or Korean were more likely to have Medicare insurance, reflecting Hawaiʻi’s demographics.^15^ Patients who identified as Pacific Islander NOS or Samoan were more likely to have commercial insurance. None of the uninsured patients in this study had any record of COVID-19 vaccination on file within the EHR (Table 1).

**Table 1.** Comparison of Demographics, COVID-19 Severity, Race and Ethnicity, and Outcomes by Insurance Type

Characteristic	Total, No. (N = 1176)	Patients, No. (%)				*P* value^a^
		Commercial (n = 458)	Medicare (n = 424)	Medicaid (n = 279)	Uninsured (n = 15)	
Sex						
Female	546 (46)	203 (44)	191 (45)	151 (54)	4 (27)	.02
Male	630 (54)	255 (56)	233 (55)	128 (46)	11 (73)	
Age, median (IQR), y	58 (41-71)	48 (37-57)	76 (69-84)	46 (31-57)	38 (35-46)	<.001
BMI, median (IQR) (n = 1160)^b^	30 (25-36)	32 (28-39)	26 (22-31)	31 (27-38)	37 (32-45)	<.001
Admission SOFA, median (IQR)	1 (0-2)	1 (0-2)	2 (1-3)	1 (0-2)	1 (0-3)	<.001
Race and ethnicity						
American Indian or Alaska Native	16	7 (2)	3 (0.7)	5 (2)	1 (7)	.17
Asian NOS	439	164 (36)	177 (42)	95 (34)	3 (20)	.06
Black	15	6 (1)	1 (<1)	8 (3)	0	.02
Chinese	66	17 (4)	38 (9)	11 (4)	0	.002
Filipino	246	91 (20)	95 (22)	58 (21)	2 (13)	.66
Hispanic	76	31 (7)	20 (5)	24 (9)	1 (7)	.30
Japanese	107	26 (6)	67 (16)	14 (5)	0	<.001
Korean	10	2 (0.4)	8 (2)	0	0	.03
Native Hawaiian	299	118 (26)	105 (25)	74 (27)	2 (13)	.67
Pacific Islander NOS	523	230 (50)	132 (31)	151 (54)	10 (67)	<.001
Samoan	156	77 (17)	25 (6)	50 (18)	4 (27)	<.001
Vietnamese	5	4 (0.9)	1 (0.2)	0	0	.29
White	311	110 (24)	125 (30)	75 (27)	2 (13)	.10
Received ≥1 vaccine dose	285	67 (15)	157 (37)	61 (22)	0	<.001
ICU transfer	130	43 (9)	52 (12)	33 (12)	2 (13)	.53
In-hospital mortality	87	18 (4)	56 (13)	12 (4)	1 (7)	<.001

Abbreviations: BMI, body mass index (calculated as weight in kilograms divided by height in meters squared); ICU, intensive care unit; NOS, not otherwise specified; SOFA, Sequential Organ Failure Assessment.

^a^ For continuous variables (age, BMI, admit SOFA), analysis of variance was used to determine statistical differences; for the remaining analyses, χ^2^ was used.

^b^ Missing BMI values were imputed with the group median (n = 8).

In multivariate analyses controlling for age, sex, BMI, comorbidities, and socioeconomic neighborhood conditions, we found no association between insurance status and ICU transfer (eg, Medicare vs commercial insurance: odds ratio [OR], 0.84; 95% CI, 0.43-1.64) or in-hospital mortality (eg, Medicare vs commercial insurance: OR, 0.85; 95% CI, 0.36-2.03) (Table 2). Furthermore, we did not find any significant association between self-identified race and ethnicity and presentation SOFA score or ICU transfer when adjusting for age, BMI, sex, comorbidities, insurance status, and socioeconomic neighborhood conditions (Table 3); of note, races and ethnicities for which there were fewer than 15 patients (eg, Black, Vietnamese) were not included in this analysis. However, in adjusted logistic regression that was univariate with respect to race and ethnicity, we found a significant positive association between Filipino self-identification and in-hospital mortality rate (OR, 1.79; 95% CI, 1.04-3.03; *P* = .03).

**Table 2.** COVID-19 Hospitalization Outcomes as a Function of Insurance Status^a^

Insurance	ICU transfer		In-Hospital Death	
	OR (95% CI)	*P* value	OR (95% CI)	*P* value
Commercial	1 [Reference]	NA	1 [Reference]	NA
Medicare	0.84 (0.43-1.64)	.61	0.85 (0.36-2.03)	.72
Medicaid	1.25 (0.74-2.08)	.39	1.11 (0.49-2.40)	.80
Uninsured	1.27 (0.19-5.05)	.77	1.99 (0.10-11.9)	.53

Abbreviations: ICU, intensive care unit; NA, not applicable; OR, odds ratio.

^a^ Adjusted for body mass index, age, sex, comorbidities shown in the eTable in Supplement 1, and proportion of households within the patient’s census geocode with below-poverty level income and/or receiving public assistance.

**Table 3.** COVID-19 Hospitalization Outcomes as a Function of Disaggregated Race and Ethnicity^a^

Race and ethnicity	ICU transfer		In-Hospital Death	
	OR (95% CI)	*P* value	OR (95% CI)	*P* value
Asian NOS	1.27 (0.85-1.87)	.24	1.55 (0.97-2.49)	.07
Chinese	0.88 (0.35-1.90)	.76	1.62 (0.69-3.41)	.23
Filipino	1.52 (0.96-2.35)	.07	1.79 (1.04, 3.03)	.03
Hispanic	1.45 (0.67-2.85)	.31	0.78 (0.22-2.10)	.66
Japanese	0.87 (0.41-1.69)	.70	1.01 (0.47-2.01)	.98
Native Hawaiian	0.70 (0.42-1.11)	.14	0.51 (0.25-0.96)	.05
Pacific Islander NOS	0.86 (0.56-1.31)	.48	0.70 (0.39-1.22)	.21
Samoan	1.39 (0.78-2.40)	.25	1.12 (0.46-2.40)	.79
White	0.75 (0.47-1.18)	.22	0.84 (0.47-1.43)	.53

Abbreviations: ICU, intensive care unit; NOS, not otherwise specified; OR, odds ratio.

^a^ Compared with the total population and adjusted for body mass index, age, sex, comorbidities shown in the eTable in Supplement 1, and proportion of households within the patient’s census geocode with below-poverty level income and/or receiving public assistance.

We separated the cohort into pre-Delta and Delta and Omicron groups based on date of admission, anticipating the possibility that different SARS-CoV-2 variants may differentially affect racial and ethnic groups. In adjusted analyses, we observed a significant association between Filipino self-identification and in-hospital mortality during the pre-Delta period (OR, 2.72; 95% CI, 1.02-7.14; *P* = .04), while patients self-identifying as Japanese were less likely to die during this period (OR, 0.19; 95% CI, 0.03-0.78; *P* = .04) (Table 4). However, Native Hawaiian patients were less likely to die during the Delta and Omicron period (OR, 0.35; 95% CI, 0.13-0.79; *P* = .02), an association that held even when adjusting for receipt of at least 1 dose of COVID-19 vaccine.

**Table 4.** Odds of In-Hospital Death by Self-Identified Race and Ethnicity by Variant Wave^a^

Race and ethnicity	Pre-Delta period (n = 459)		Delta and Omicron Period (n = 717)				
	OR (95% CI)	*P* value	No vaccine adjustment		Vaccine-adjusted		
			OR (95% CI)	*P* value	OR (95% CI)	*P* value
Asian NOS	1.70 (0.74-3.91)	.21	1.39 (0.75-2.54)	.29	1.45 (0.78-2.71)	.24
Chinese	2.18 (0.50-8.19)	.27	1.36 (0.43-3.57)	.56	1.66 (0.52-4.42)	.35
Filipino	2.72 (1.02-7.14)	.04	1.42 (0.69-2.78)	.32	1.36 (0.65-2.73)	.40
Hispanic	1.45 (0.18-7.80)	.69	0.56 (0.09-2.03)	.45	0.56 (0.09-2.09)	.46
Japanese	0.19 (0.03-0.78)	.04	2.27 (0.92-5.25)	.06	2.30 (0.92-5.45)	.06
Native Hawaiian	1.01 (0.30-2.90)	.99	0.35 (0.13-0.79)	.02	0.34 (0.13, 0.77)	.02
Pacific Islander NOS	0.62 (0.20-1.73)	.38	0.84 (0.41-1.69)	.64	0.76 (0.37-1.54)	.46
Samoan	0.38 (0.02-2.18)	.37	1.79 (0.66-4.40)	.22	1.61 (0.58-4.00)	.33
White	0.82 (0.28-2.21)	.71	0.74 (0.35-1.45)	.40	0.75 (0.35-1.48)	.42

Abbreviations: NOS, not otherwise specified; OR, odds ratio.

^a^ Compared with the total population and adjusted for body mass index, age, sex, comorbidities shown in the eTable in Supplement 1, and proportion of households within the patient’s census geocode with below-poverty level income and/or receiving public assistance.

When evaluating vaccination status after June 10, 2021 (encompassing the Delta and Omicron waves), when 65% of Hawaiʻi residents had received at least 1 dose of a COVID-19 vaccine, we found a positive association between Medicare insurance and vaccine receipt (OR, 1.85; 95% CI, 1.07-3.21; *P* = .03), indicating that Medicare patients were more likely to be vaccinated compared with commercially insured patients (Table 5). We found no significant association of self-identified race and ethnicity with vaccination status in adjusted analyses. We also found a significant positive association between vaccination status and SOFA score at the time of admission: vaccinated patients presented with SOFA scores 0.376 points higher (indicating greater organ dysfunction) than their unvaccinated counterparts of the same age and insurance status (*P* = .05). Vaccination status was also associated with reduced ICU admission (OR, 0.40; 95% CI, 0.21-0.70; *P* = .002) as well as in-hospital mortality (OR, 0.42; 95% CI, 0.21-0.79; *P* = .01).

**Table 5.** Vaccination Status as a Function of Insurance Status After June 10, 2021^a^

Insurance	Vaccinated (n = 707)	
	OR (95% CI)	*P* value
Commercial	1 [Reference]	NA
Medicare	1.85 (1.07-3.21)	.03
Medicaid	1.36 (0.89-2.07)	.15
Uninsured	NA^b^	NA^b^

Abbreviations: NA, not applicable; OR, odds ratio.

^a^ Adjusted for body mass index, age, and sex.

^b^ Sample insufficient for logistic regression, excluded.

## Discussion

In this cohort study, we found that there were no differences in ICU transfer or in-hospital mortality associated with insurance type among a large cohort of patients hospitalized for COVID-19 at a single tertiary care center. Furthermore, in secondary analyses using disaggregated self-identified race and ethnicity data, we observed an association between race and insurance status, with a higher proportion of Chinese, Japanese, and Korean patients receiving Medicare insurance and higher proportions of Pacific Islander and Samoan patients covered by commercial insurance. In analyses adjusting for age, sex, BMI, comorbidities, socioeconomic neighborhood conditions, and insurance status, we identified significant differences in outcomes for COVID-19 hospitalization associated with race and ethnicity, with Filipino patients more likely to die in the hospital and Japanese patients less likely to die in the hospital during the pre-Delta period and Native Hawaiians less likely to die in the hospital during the Delta and Omicron period. Finally, we found that receipt of at least 1 COVID-19 vaccination was associated with reduced ICU transfer and in-hospital mortality among all races and ethnicities.

Patients with Medicare insurance are necessarily older and have more medical comorbidities than those covered by Medicaid or commercial insurance and thus would be expected to sustain poor outcomes without age or comorbidity adjustment.^16^ Males are more likely to be hospitalized and die with COVID-19 (consistent with our observations), and patients with higher BMIs are known to have poorer outcomes from COVID-19.^17,18^ However, when adjusting for age, BMI, sex, and medical comorbidities, we observed no difference in outcomes between patients of differing insurance status. At the study hospital, similar care was provided for inpatients regardless of insurance status, and thus it is reassuring that no difference in outcomes was identified. Within the Kaiser Permanente integrated care delivery model, the covered benefits (for both inpatient and outpatient health care) are similar across Kaiser commercial insurance products, Kaiser Medicare, and Kaiser Medicaid. However, when considering the disparities in social determinants of health among these groups, our finding of similar outcomes regardless of insurance status was unexpected. This observation serves not only as an internal proof-of-concept of the equity of the Kaiser Permanente model but also more broadly as an endorsement of universal health care coverage as a means of achieving health equity.^19^ Recent analyses support the provision of universal health care as a form of pandemic preparation that ultimately saves lives.^11^ Although prior studies suggested that patients insured through Medicaid programs have worse outcomes when hospitalized for sepsis,^20^ our data indicate that patients with Medicaid fared as well as those with commercial or Medicare insurance (even when adjusting for socioeconomic neighborhood conditions), suggesting that the quality of the Medicaid coverage itself may also be important.^12^

Disaggregation of Asian, Native Hawaiian, and Pacific Islander race and ethnicity data is a crucial first step to addressing health disparities among high-risk populations. Seminal analysis by the Hawaiʻi Department of Health (HDOH) revealed significant differences in incidence and outcome of COVID-19 among different populations during the first year of the pandemic, although the lack of age-adjustment limited their ability to control for this critical driver of COVID-19 mortality.^7^ In our work (as in the HDOH analysis), Filipino patients had significantly higher risks of death during hospitalization throughout the pandemic, particularly during the pre-Delta period. That this worse outcome occurred even when controlling for type of insurance suggests that provision of health insurance alone will be insufficient to improve outcomes and necessitates exploring other social determinants of health. De la Cruz et al^21^ identified numerous gaps in social, health, and financial service infrastructures that may explain worse outcomes among Filipino patients the COVID-19 pandemic, including language barriers, food instability, financial insecurity, a high proportion of frontline health workers or tourism industry employees, and higher rates of chronic disease. Other potential considerations include evolving distrust in public health recommendations, vaccine hesitancy, living situations (eg, congregate or multigenerational), and culture-specific concepts of health.^22,23,24^ In contrast, Native Hawaiian patients fared better in our analyses, with lower rates of in-hospital mortality, particularly during the Delta and Omicron period. While these data are similar to those collected by the HDOH, they contrast substantially with data on Native Hawaiian individuals and Pacific Islander individuals living in states other than Hawaiʻi.^25,26^ Given that Native Hawaiian individuals have a number of social and health disparities associated with increased risk for poor outcomes during COVID-19, possible explanations for this counterintuitive outcome include specific efforts targeting vaccination to Native Hawaiian individuals (with results not captured in this dataset), delayed first cases of COVID-19 (as indicated by HDOH data) thereby allowing provision of newly approved COVID-19 therapies, and an overall benefit of having health insurance within this cohort.^22,26^ Finally, our observation that Japanese patients had better outcomes in the pre-Delta period but worse outcomes during the Delta and Omicron period merits further investigation, particularly given that Japanese patients (like Native Hawaiian patients) were less likely to be diagnosed with COVID-19 early in the pandemic.^7^

While our vaccine data were incomplete, we did observe a higher vaccination rate among Medicare patients during the Delta and Omicron period (when >65% of Hawaiʻi residents had received ≥1 vaccination) compared with other insurance designations; this is unsurprising, given the effort to vaccinate individuals at high risk of adverse outcomes during the initial vaccination campaign.^26^ In addition, we observed a significant association between vaccination and reduced ICU admission and death among patients of all races and ethnicities. Interestingly, patients who received at least 1 vaccine presented with a higher median SOFA score on admission. As SOFA score is a validated measure of acute morbidity of critical illness at the population level, this observation may reflect health care practitioners applying a higher acuity threshold for hospital admission in vaccinated patients compared with unvaccinated patients, or it may reflect higher rates of vaccination in patients with more significant comorbidities.^27^ However, in spite of having a higher median SOFA score, vaccinated patients had better outcomes, thus reinforcing the importance of vaccination in the pandemic.

### Limitations

Limitations of this study include the inherent variability in collecting self-identified race and ethnicity data for entry into the EHR, with a 2022 study^6^ suggesting that this approach underestimates identification of Native Hawaiian and Pacific Islander patients in COVID-19 hospitalizations.^6^ Furthermore, the vaccine data are incomplete, given that patients may have received vaccines at other sites not captured in the EHR. In addition, too few uninsured patients were admitted to this hospital to evaluate for associations with race and ethnicity or vaccine status in our analysis. In addition, observations from a single health care center are not representative of all state residents or members of a specific race or ethnicity: many Native Hawaiian individuals live in regions of Oahu served by other hospitals, making it less likely that they would be brought by ambulance to the study hospital, unless they were transferred because of having Kaiser Permanente insurance. Residents of these regions experience well-documented shortages of health care services and decreased life expectancies, suggesting that Native Hawaiian individuals in our study may have certain advantages over those elsewhere in the state, a possibility that will be explored in future work.^28,29^ Our observation that there were such significant outcome disparities among Filipino individuals, consistent with analysis by the HDOH, also merits further exploration and potential targeting of resources to improve these outcomes.

## Conclusions

The findings of this cohort study suggest that health insurance coverage provided in an integrated care delivery model may be associated with mitigating the effects of disparities in social determinants of health and clearly indicates that outcomes from COVID-19 hospitalization vary substantially by race and ethnicity, especially when applying disaggregation to the otherwise monolithic category of Asian, Native Hawaiian, and Pacific Islander. Efforts to expand insurance coverage and to understand the impacts of disease on disaggregated populations should be important priorities both in preparing for the next pandemic and for assuring health equity in the US.

## Notes

Supplement 1. **eTable.** Participant Comorbidities by Insurance Status

Supplement 2.
Data Sharing Statement
