# Supplementary material for: COVID-19 Hospitalization in Hawaiʻi and Patterns of Insurance Coverage, Race and Ethnicity, and Vaccination
Source: JAMA Netw Open. 2024 May 1;7(5):e243696. doi: 10.1001/jamanetworkopen.2024.3696 (PMC11063802; doi:10.1001/jamanetworkopen.2024.3696)
Supplement: Supplement 1. — eTable. Participant Comorbidities by Insurance Status [file jamanetwopen-e243696-s001.pdf]

Supplemental Online Content

Santi BM, Verhoef PA. COVID-19 hospitalization in Hawai‘i and patterns of insurance coverage, race and ethnicity, and vaccination. *JAMA Netw Open*. 2024;7(5):e243696. doi:10.1001/jamanetworkopen.2024.3696

eTable. Participant Comorbidities by Insurance Status

This supplemental material has been provided by the authors to give readers additional information about their work.

© 2024 Santi BM et al. *JAMA Network Open.*

eTable. Participant Comorbidities by Insurance Status

Comorbidity, n (%)	Total n=1176	Commercial n=458	Medicare n=424	Medicaid n=279	Uninsured n=15	p-value[a]
Asthma	271 (23)	91 (20)	96 (23)	82 (29)	2 (13)	0.02
Cancer	99 (8)	22 (5)	67 (16)	9 (3)	1 (7)	**<0.001**
Stroke	67 (6)	10 (2)	38 (9)	18 (7)	1 (7)	**<0.001**
Chronic Kidney Disease	390 (33)	71 (16)	249 (59)	68 (24)	2 (13)	**<0.001**
Chronic Lung Disease	115 (10)	19 (4)	84 (20)	12 (4)	0 (0)	**<0.001**
Chronic Liver Disease	148 (13)	51 (11)	52 (12)	41 (15)	4 (27)	0.19
Cystic Fibrosis	87 (7)	29 (6)	35 (8)	23 (8)	0 (0)	0.44
Diabetes	563 (48)	191 (42)	236 (56)	130 (47)	6 (40)	**<0.001**
Cardiovascular Disease	371 (32)	65 (14)	231 (55)	72 (26)	3 (20)	**<0.001**
HIV	1 (0.1)	0 (0)	1 (0.2)	0 (0)	0 (0)	0.62
Organ Transplant Recipient	6 (0.5)	1 (0.2)	5 (1)	0 (0)	0 (0)	0.11
Tuberculosis	2 (0.2)	0 (0)	2 (0.5)	0 (0)	0 (0)	0.31
Chronic Corticosteroid Use	70 (6)	10 (2)	48 (11)	11 (4)	1 (7)	**<0.001**
Pregnancy	81 (7)	36 (8)	0 (0)	45 (16)	0 (0)	**<0.001**

[a] Pearson chi-squared test.

© 2024 Santi BM et al. *JAMA Network Open.*
