# Supplementary material for: COVID-19 Hospitalization in Hawaiʻi and Patterns of Insurance Coverage, Race and Ethnicity, and Vaccination
Source: JAMA Netw Open. 2024 May 1;7(5):e243696. doi: 10.1001/jamanetworkopen.2024.3696 (PMC11063802; doi:10.1001/jamanetworkopen.2024.3696)
Supplement: Supplement 2. — Data Sharing Statement [file jamanetwopen-e243696-s002.pdf]

Data Sharing Statement

Santi. COVID-19 Hospitalization in Hawaiʻi and Patterns of Insurance Coverage, Race and Ethnicity, and Vaccination. *JAMA Netw Open*. Published May 01, 2024.
doi:10.1001/jamanetworkopen.2024.3696

Data
Data available: No

Additional Information
Explanation for why data not available: Data sharing requests will be considered if data to be shared is de-identified and approved by Hawaii Permanente Medical Group.
